# Frailty from conceptualization to action: the biopsychosocial model of frailty and resilience

**DOI:** 10.1007/s40520-022-02337-z

**Published:** 2023-03-22

**Authors:** M. Cristina Polidori, Luigi Ferrucci

**Affiliations:** 1 Ageing Clinical Research, Department II of Internal Medicine and Center for Molecular Medicine Cologne, Faculty of Medicine, University Hospital Cologne, University of Cologne, Cologne, Germany; 2 Cologne Excellence Cluster on Cellular Stress-Responses in Aging-Associated Diseases (CECAD), Faculty of Medicine, University Hospital Cologne, University of Cologne, Cologne, Germany; 3 Longitudinal Studies Section, Translational Gerontology Branch, National Institute on Aging, National Institutes of Health, Baltimore, MD USA

The term “frailty” was first introduced in the geriatric literature in 1982 [1], and since then, more than 22,000 scientific articles have appeared in PubMed that directly or indirectly address age-related frailty. The scientific search for a definition of frailty and a better understanding of its causes and consequences substantially increased when Linda Fried first described frailty as a quantifiable–operationalized-clinical entity using data from the Cardiovascular Health Study [2]. This first definition was followed most noticeably by the “frailty index” proposed by Kenneth Rockwood [3]. The development of these two operational definitions was paralled by many additional instruments which attempted to be enough parsimonious to be used with the time constrains of clinical practice or to explore enough details to capture the complexity of biological, physiological and environmental elements that influence the overall vulnerability that develops with aging. Physical/cognitive frailty is now widely accepted as a geriatric syndrome and research in this field has provided substantial knowledge on risk factors, mechanisms, as well as short and long-term consequences. In spite of growing discussion on frailty and the fact that this condition is associated with poor health and adverse outcomes including falls, multimorbidity, disability, death and poor quality of life [4, 5], this concept remains substantially theoretical.

Healthcare practitioners—within and outside the geriatric specialty—understand the need for a frailty definition that is “simple but no simpler” [6]. However, unless we can provide evidence that screening frailty—even with some “imperfect” tool—offers clinical advantage, the science of frailty will remain pure speculation.

And yet, reducing the burden of frailty and improving health outcomes in those who are already frail are not an option but rather an urgent necessity. The healthcare system, already disarmed against the consequences of inadequate management of 703 million persons aged 65 years or over in the world in 2019, will inevitably suffer even more challenged by the estimated doubling of this figure to 1.5 billion in 2050 (https://www.rcplondon.ac.uk/guidelines-policy/hospitals-edge-time-action): the share of world population aged 65 years or more increased from 6 in 1990 to 9% in 2019 and is projected to rise further to 16% by 2050, so that one in six people in the world will be aged 65 years or over.

A wide examination of the literature shows that frailty no longer belongs to the field of “eminence-based medicine” given the large body of mechanistic and clinical research appeared in the literature of the last few years. Screening for and correct diagnosis of frailty in parallel but independently of the diagnosis of chronic disease is beneficial both to medical institution and to patients and their families. Unfortunately, little of this science is translated into the organization of a stronger connection between the medical and social support systems, in part because such organization is based on political will rather than on scientific evidence.

As researchers and geriatricians, we can see at least two possible paths to overcome this impasse. First, we need to emphasize the evidence accumulated so far and shift our attention from improving the operational definition to expand its implementation in clinical thinking and management. Increased susceptibility to stressors and impaired functional reserves are the fundamental characteristics of persons with frailty, a dangerous condition associated, though not coincident with increasing chronological age [7]. We believe that understanding the mechanistic causes of frailty at a biological level may represent a substantial step toward the identification of interventions that effectively reduce the burden of this syndrome in the older population. However, it is important that biologists and clinicians join forces in this research endeavor, so that a translation perspective is maintained in the design and execution of any experiment or study. This is a second feasible way to put principles into practice for an efficacious integration of frailty diagnosis in clinical decision-making.

It is with this idea of basic-biologic and clinical integration that we launch this collection of articles that address frailty-related areas of science, including “hallmarks”, biomarkers and clocks, phenotypes, ICD definitions, and ongoing drug-testing studies. The role of senescence [8] and frailty [9] for COVID-19 lethality, circadian rhythm [10] and insomnia [11] for frailty onset and progression, as well as the relationship between physiology of aging, frailty and hypertension [12], chronic kidney disease [13] and radiotherapy [14] are presented here. Among other international researchers, students of the EITHealth Ageing PhD School participating to the 2021 edition of the advanced Innovation&Entrepeneurship course “Frailty in real life and its translational implications” (CECAD: EIT Health Ageing PhD School (uni-koeln.de)) accepted the challenge of participating to the additional accredited course on scientific writing and present their research in the present collection. For the planning of manuscript drafting on their individual research topics, the students were encouraged to adopt a strategy embracing complexity—the dominant characteristic of the aging process though systematically avoided in the clinic and for traditional research purposes—thereby looking at their experiments in the context of real life. This seemingly academic exercise resulted into ground-breaking results that pointed to unresolved questions, missing links, misleading speculations, and weak assumptions on frailty and its biology [8–15]. For this reason, the articles include a nourished “research outlook” section and show the potential of including traditionally uncomfortable parameters, such as heterogeneity, dynamics, and multifactoriality in experimental protocols.

From the point of view of mentoring in the field of frailty, there is urgent need to 1) use computational biology to plan studies on complex models, not only to analyze big existing data; 2) explore further hallmarks and biomarkers that are more physiologically relevant and can be directly translated to care; 3) include multifactorial robustness and residual resilience as fundamental actors in frailty trajectories; 4) develop accurate instruments to capture early frailty stages, frailty-robustness dynamics overtime and, most importantly, the grade of vulnerability prior to acute stressor before the health crisis that precipitates the access to the health care system.

After several decades spent to define monodimensional DRG codes useful for the medicine of acute conditions in robust patients, this system is currently applied to complex, vulnerable patients with chronic conditions treated with drugs tested in completely different systems. The description of the construct of frailty as a distinct clinical entity enabled a better personalized medicine (Fig. 1) but at the same time unveiled the invisible part of the iceberg—Biological Age to be measured by individual multidimensional frailty in the energy-based, dynamic oscillatory balance between damage and repair. In this sense, frailty emerges as the very core of (geriatric) medicine more than a syndrome; in fact, a state characterized by a condition in which residual resilience is so curtailed that even the challenges of living a normal life induce stress and accumulation of damage [16]. From this perspective, building resilience (intrinsic, environmental or both) is the only way to counteract frailty. This is the paradigm shift needed in medicine 2020–2050 and is therefore the urgent quest for a research agenda consistently resources-oriented.

**Fig. 1:**
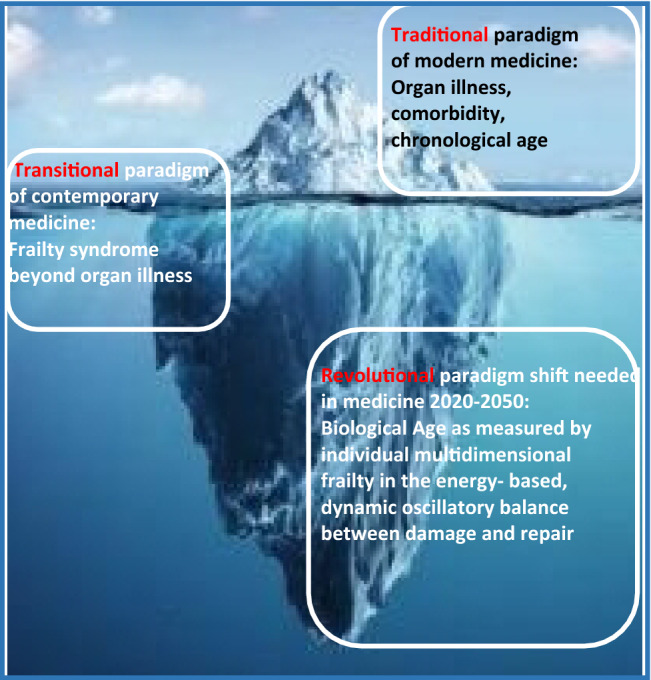
The frailty and biological age iceberg. The *traditional* paradigm of medicine bases on the biological model of disease, established when the large majority of diseases was of acute nature. This model prevalently addresses morbidity and chronological age for clinical decision-making and is strongly associated with high healthcare costs and poor quality of life. With the rapid increase and highest prevalence of chronic noncommunicable diseases of the past decades, however, contemporary medicine is more and more adopting a multidimensional, *biopsychosocial* concept of health and disease. As a consequence of this *transitional* paradigm, the ultimate *revolutional* shift is needed now and in the near future: frailty as a surrogate marker of biological age is recognized as the unavoidable concept to improve health trajectories with advancing age. However, healthcare systems still struggle to adopt its diagnosis and treatment systematically. Therefore, the paradigm shift needed comprehends joint efforts between researchers and physicians to assess frailty and use the biopsychosocial model of biological age for sustainable interventions in clinical routine

## References

[1] Brocklehurst JC, Robertson D, James-Groom P (1982). Skeletal deformities in the elderly and their effect on postural sway. J Am Geriatr Soc.

[2] Fried LP, Tangen CM, Walston J, Cardiovascular Health Study Collaborative Research Group (2001). Frailty in older adults: evidence for a phenotype. J Gerontol A Biol Sci Med Sci.

[3] Mitnitski AB, Mogilner AJ, Rockwood K (2001). Accumulation of deficits as a proxy measure of aging. Sci World J.

[4] Dent E, Martin FC, Bergman H (2019). Management of frailty: opportunities, challenges, and future directions. Lancet.

[5] Hoogendijk EO, Afilalo J, Ensrud KE (2019). Frailty: implications for clinical practice and public health. Lancet.

[6] Chetrit J, Mendis N, Afilalo J (2021). Frailty: as simple as possible, but no simpler. Circ Cardiovasc Qual Outcomes.

[7] Zampino M, Polidori MC, Ferrucci L (2022). Biomarkers of aging in real life: three questions on aging and the comprehensive geriatric assessment. GeroScience.

[8] Carvalhas-Almeida C, Cavadas C, Álvaro AR (2023) The impact of insomnia on frailty and the hallmarks of aging. Aging Clin Exp Res 35:253–269. 10.1007/s40520-022-02310-w 10.1007/s40520-022-02310-w PMC9895045 36583849

[9] Liu P, Li Y, Li S et al (2023) Serum progranulin as a potential biomarker for frailty in Chinese older adults. Aging Clin Exp Res 35:399–406. 10.1007/s40520-022-02318-2 10.1007/s40520-022-02318-2 36562981

[10] Ribeiro RFN, Pereira D de Almeida LP et al (2022) SIRT1 activation and its circadian clock control: a promising approach against (frailty in) neurodegenerative disorders. Aging Clin Exp Res 34:2963–2976. 10.1007/s40520-022-02257-y 10.1007/s40520-022-02257-y 36306110

[11] Zhang H, Hao M, Li Y et al (2022) Glomerular filtration rate by different measures and albuminuria are associated with risk of frailty: the Rugao Longitudinal Ageing Study. Aging Clin Exp Res 34:2703–2711. 10.1007/s40520-022-02245-2 10.1007/s40520-022-02245-2 36260213

[12] Zhu Y, Sealy MJ, Jager-Wittenaar H et al (2022) Frailty and risk of hospitalization from COVID-19 infection among older adults: evidence from the Dutch Lifelines COVID-19 Cohort study. Aging Clin Exp Res 34:2693–2702. 10.1007/s40520-022-02268-9 10.1007/s40520-022-02268-9 PMC9572827 36244048

[13] Qipo O, Debain A, Bautmans I et al (2022) The influence of hypertension management on frailty prevention among older persons aged 65 and over: a systematic review. Aging Clin Exp Res 34:2645–2657. 10.1007/s40520-022-02247-0 10.1007/s40520-022-02247-0 36195809

[14] Rodrigues ED, Gonsalves D, Teixeira L et al (2022) Frailty—the missing constraint in radiotherapy treatment planning for older adults. Aging Clin Exp Res 34:2295–2304. 10.1007/s40520-022-02200-1 10.1007/s40520-022-02200-1 36056189

[15] Müller-Werdan U, Polidori MC, Simm A. On Frailty and accelerated aging during SARS-Cov-2: Senescence. Aging Clin Exp Res 34 in press 10.1007/s40520-023-02364-4 PMC10025062 36935472

[16] Ferrucci L, Gonzalez-Freire M, Fabbri E (2020). Measuring biological aging in humans: a quest. Aging Cell.

